# Non-Target-Site Resistance to Herbicides: Recent Developments

**DOI:** 10.3390/plants8100417

**Published:** 2019-10-15

**Authors:** Mithila Jugulam, Chandrima Shyam

**Affiliations:** Department of Agronomy, Kansas State University, Manhattan, KS 66506, USA

**Keywords:** non-target-site resistance, herbicide mode of action, co-existence, environmental conditions

## Abstract

Non-target-site resistance (NTSR) to herbicides in weeds can be conferred as a result of the alteration of one or more physiological processes, including herbicide absorption, translocation, sequestration, and metabolism. The mechanisms of NTSR are generally more complex to decipher than target-site resistance (TSR) and can impart cross-resistance to herbicides with different modes of action. Metabolism-based NTSR has been reported in many agriculturally important weeds, although reduced translocation and sequestration of herbicides has also been found in some weeds. This review focuses on summarizing the recent advances in our understanding of the physiological, biochemical, and molecular basis of NTSR mechanisms found in weed species. Further, the importance of examining the co-existence of TSR and NTSR for the same herbicide in the same weed species and influence of environmental conditions in the altering and selection of NTSR is also discussed. Knowledge of the prevalence of NTSR mechanisms and co-existing TSR and NTSR in weeds is crucial for designing sustainable weed management strategies to discourage the further evolution and selection of herbicide resistance in weeds.

## 1. Introduction

Herbicide use is indispensable in modern agriculture as it offers exceptional tool for weed management and also facilitates no-till crop production to conserve soil and moisture. However, repeated field applications of herbicides with the same mechanism of action resulted in the selection of herbicide-resistant weeds. The Weed Science Society of America (http//www.wssa.net) defines herbicide resistance as the inherited ability of a plant to survive and reproduce following exposure to a dose of herbicide normally lethal to the wild type. Under continuous selection pressure, i.e., the repeated use of herbicides with the same mode of action, the resistant plants increase in frequency over time, resulting in the domination by individuals resistant to that herbicide. In addition to the selection pressure of herbicides, biological and genetic factors of weed species, properties of herbicides, and agronomic practices also play an important role in the evolution and spread of herbicide resistance [1]. Biological characteristics of highly troublesome weeds, including prolific seed production, high germination percentage, a wide window of emergence, seed dispersal, and longevity, help to maintain a high frequency of resistant individuals in the population. Genetic factors, such as natural mutations conferring herbicide resistance, inheritance of herbicide-resistant genes in the weed population, and fitness cost of resistance genes in the presence or absence of the herbicide, also play an important role in the evolution and spread of herbicide resistance [2,3].

## 2. Mechanisms of Herbicide Resistance

A key aspect in predicting the evolutionary trajectory of herbicide-resistance traits is understanding the mechanism(s) of herbicide resistance. Mechanisms of herbicide resistance in weeds can be broadly classified into target-site resistance (TSR) and/or non-target-site resistance (NTSR). The TSR mechanisms largely involve mutation(s) in the target site of action of an herbicide, resulting in an insensitive or less sensitive target protein of the herbicide [1]. In such cases, the TSR is primarily determined by monogenic traits [3]. Additionally, TSR can also evolve as a result of the over-expression or amplification of the target gene [4]. NTSR mechanisms include reduced herbicide uptake/translocation, increased herbicide metabolism, decreased rate of herbicide activation, and/or sequestration [5]. Metabolism-based NTSR involves the increased activity of enzyme complexes such as esterases, cytochrome P450s (CYP450s), glutathione S-transferases (GSTs), and/or Uridine 5’-diphospho (UDP)-glucosyl transferases [1]. NTSR, especially if it involves herbicide detoxification by these enzymes, is usually governed by many genes (polygenic) and may confer resistance to herbicides with completely different modes of action [3,6]. However, monogenic inheritance of NTSR has also been reported in several herbicide-resistant weeds [7,8,9]. The evolution of NTSR via herbicide detoxification is a serious threat to weed management as it can bestow resistance to multiple herbicides, leaving limited herbicide options for weed control, as well as potential resistances to herbicides not yet commercially available [10]. Comprehensive information on the evolution of TSR-based resistance in weeds are discussed elsewhere in this special issue. In this review recent advances in understanding the mechanisms of NTSR to herbicides with different modes of action are discussed.

## 3. Known NTSR Mechanisms in Weed Species for Different Herbicide Modes of Actions

### 3.1. Acetyl CoA Carboxylase (ACCase)-Inhibitors

ACCase is a crucial enzyme that catalyzes the formation of malonyl CoA via the carboxylation of acetyl CoA while using bicarbonate as the source of carbon [11]. Malonyl CoA is needed for de novo fatty acid biosynthesis, and thus, is essential for plant survival. ACCase-inhibitors impede malonyl CoA formation in sensitive grass species, ultimately leading to plant death [11,12]. These herbicides are used as important post-emergence options for managing grass weeds in dicotyledonous crops. To date, 48 weeds have been reported to have evolved resistance to these herbicides [13] via both TSR and NTSR mechanisms. Predominantly, TSR has been reported as the leading mechanism, caused by amino acid substitutions in the carboxyl transferase domain of the ACCase enzyme [14,15].

Metabolic resistance to ACCase-inhibiting herbicides has been documented in Asia minor bluegrass [16], barnyard grass [17], blackgrass [18], Italian ryegrass [19,20], Japanese foxtail [21], rigid ryegrass [22,23,24], and wild oat [25]. In the majority of these cases, enhanced metabolism mediated by CYP450s was reported. For instance, rapid degradation of diclofop-methyl was observed in rigid ryegrass populations from Australia [22,24]. Interestingly, exposure to low doses of diclofop-methyl acid application rapidly selected for metabolic resistance in rigid ryegrass [26]. Moreover, the metabolites produced in these resistant plants were found to be similar to those in wheat formed via ring hydroxylation and sugar conjugation [26]. This result suggests that in resistant grasses, the metabolism of ACCase-inhibitors occurs through a wheat-like detoxification pathway mediated by CYP450s [25,26]. Studies involving CYP450 or GST inhibitors, such as malathion and piperonyl butoxide (PBO), have been used to indicate the involvement of these detoxification systems. The organophosphate insecticide, malathion can decrease the rate of metabolism and increase the metabolic half-life of herbicides by inhibiting CYP450-based hydroxylation in corn [27,28]. Pre-treatment with CYP450 inhibitors like PBO or malathion has been shown to reduce resistance to ACCase-inhibitors in Asia minor bluegrass [16] and Japanese foxtail [21], indicating the role of CYP450s in enhancing metabolism in these resistant weeds. Conversely, pre-treatment with 2,4-D, a CYP450 inducer, increased the rate of metabolism of diclofop-methyl in susceptible rigid ryegrass populations [29]. Apart from CYP450s, involvement of GSTs and glucosyltransferases (GTs) have also been documented to govern the metabolic resistance to ACCase-inhibitors. Transcriptome analysis of diclofop-resistant rigid ryegrass, led to the identification of four contigs, including two *CYP450s*, one *GT*, and one nitronate monooxygenase (*NMO*) as potential candidate genes for metabolic resistance to diclofop [24]. Similarly, researchers have reported greater GST activity in resistant plants following ACCase-inhibitor application [16,17,30].

### 3.2. Acetolactate Synthase (ALS)-Inhibitors

ALS inhibitors were first commercialized in 1982, and by 1998, the number of weed species with resistance to this group of herbicides had surpassed other herbicides [31]. These herbicides, also referred as acetohydroxy acid synthase (AHAS) inhibitors, inhibit ALS or AHAS enzyme, which is vital for the biosynthesis of branched-chain amino acids isoleucine, leucine, and valine [32]. In general, these are broad-spectrum, post-emergence herbicides used for controlling weeds in a variety of crops like wheat and soybean. However, few ALS inhibitors, such as trifloxysulfuron, are also used as pre-emergence options to control weeds. Currently, resistance to ALS inhibitors is reported in 161 weeds globally [13]. TSR caused by single amino acid substitutions has been reported in most of these ALS-inhibitor-resistant weeds. Until recently, detection of these mutations have highlighted more importance on identifying TSR mechanisms compared to NTSR, even though they can co-exist in the same population [33,34]. However, the identification of plants lacking mutations in the ALS domain and surviving herbicide application has led researchers to focus on elucidating the NTSR mechanisms.

Enhanced metabolism conferring resistance to ALS inhibitors has been documented in several grass and broadleaf weeds, such as barnyard grass [35], common waterhemp [36], Palmer amaranth [37], rice barnyard grass [38,39], rigid brome [40], short awn foxtail (*Alopecurus aequalis*) [41,42], and water chickweed (*Myosoton aquaticum*) [33]. Numerous studies have also elucidated the molecular basis of metabolic resistance to ALS inhibitors. Though genes involved in metabolic resistance can be different depending on the weed species and history of herbicide application [43], most of these studies have predominantly identified multiple *CYP450* genes that are either constitutively expressed or upregulated following ALS inhibitor application [38,41,44]. For example, the mechanism of mesosulfuron-methyl resistance in short awn foxtail was studied and two *CYP450* genes, i.e., CYP94A1 and CYP71A4, were identified to be constitutively overexpressed in the resistant plants [41]. In a similar study, two *CYP450* genes, i.e., *CYP81A12* and *CYP81A21*, were identified as candidate genes conferring resistance to bensulfuron-methyl and penoxsulam in rice barnyard grass [38]. Several *CYP450* genes mediating NTSR to ALS inhibitors have been identified in water chickweed [44], ryegrass [45], flixweed [46], and blackgrass [18,47]. In addition to CYP450s, involvement of GSTs, GTs, and ATP-binding cassette (ABC) transporters have also been reported [42,44,45,46]. For instance, in ALS-inhibitor-resistant water chickweed, four genes—including three *CYP450s* (having homology to *CYP734A1*, *CYP76C1*, and *CYP86B1*) and an *ABC* transporter (having homology to *ABCC10*)—were identified as being highly expressed in all resistant plants [44]. Another commonly used procedure to test the CYP450 mediated metabolic resistance to ALS inhibitors has been the increase in sensitivity upon pre-treatment with CYP450 inhibitors, such as PBO, phorate, and malathion. Such increased sensitivity was observed in rigid ryegrass [48], short awn foxtail [41], Palmer amaranth [37], common waterhemp [36], barnyard grass [35], and rigid brome [40]. Malathion application also reversed 2,4-D-induced protection against chlorsulfuron in susceptible rigid ryegrass from Australia, suggesting the involvement of CYP450s in metabolizing chlorsulfuron [29].

### 3.3. Synthetic Auxinic Herbicides

Synthetic auxinic herbicides (SAH) are known to mimic the natural plant hormone, indole 3-acetic acid (IAA) [49]. These auxin analogs are mostly used for controlling broadleaf weeds in monocot crops, except quinclorac and quinmerac, which are known to have some grass activity [50]. Despite being introduced as early as 1945, the evolution of resistance to SAH has been slow, and so far, 39 weeds are reported to have developed resistance [13]. In the majority of weeds, NTSR mechanism(s) via (i) reduced uptake, (ii) decreased translocation, and (iii) increased metabolism has been documented. The reduced uptake of SAH is often affected by the properties of the leaf cuticle or other structural barriers that prevent absorption of the herbicide into mesophyll after herbicide application [51]. However, reduced uptake is a minor mechanism and has been shown to impart resistance in fewer weeds, such as ground ivy [51] and prickly lettuce [52]. However, reduced translocation resulting in the decreased movement of SAH to the site of action is common. Such a reduction in translocation was reported in several weed species, such as wild radish [53], oriental mustard [54], corn poppy [55], and prickly lettuce [52]. For instance, reduced translocation was observed in oriental mustard where approximately 77% of 2,4-D (2,4-dichlorophenoxyacetic acid) was retained in the treated leaves of resistant plants compared to 32% in susceptible plants at 72 h after treatment (HAT) [54]. In another study, the application of auxin efflux inhibitors 1-naphthylphthalamic (NPA) and 2,3,5-triiodobenzoic acid (TIBA) via roots of 2,4-D-susceptible wild radish plants significantly inhibited the translocation of 2,4-D out of the treated leaves, mimicking the 2,4-D-resistant wild radish [53]. Application of the same inhibitors did not affect the translocation of 2,4-D in the resistant biotype, suggesting alteration of the activity of ABC-transporters present in the plasma-membrane that usually facilitate the long-distance transport of 2,4-D [53]. MCPA (2-methyl-4-chlorophenoxyacetic acid) resistance in wild radish from Australia has been attributed to the rapid translocation to the roots [56]. At 48 HAT, a significantly lower amount of MCPA was recovered from resistant plants compared to susceptible plants, suggesting a possible root exudation of MCPA out of the plants [56]. Rapid metabolism of SAH is another major NTSR mechanism reported in several dicot weed species, where similar to the naturally tolerant monocot species, detoxification of herbicides occurs via ring-hydroxylation followed by conjugation, mediated by CYP450s. Such rapid detoxification of SAH has been reported in common waterhemp [36,57] and corn poppy [58]. In 2,4-D-resistant common waterhemp, 2,4-D was found to metabolize at a much faster rate compared to the susceptible plants, resulting in a lower metabolic half-life of 2,4-D [57]. In two 2,4-D-resistant corn poppy populations from Spain, enhanced metabolism was reported [58]. Two hydroxy metabolites were detected in the roots and shoots of the resistant plants, but not in the susceptible plants, suggesting a possible enhanced metabolism of herbicide due to CYP450-based hydroxylation in resistant plants [58]. Increased sensitivity of SAH-resistant biotypes was observed when pre-treated with CYP450-inhibitor malathion followed by herbicide application [36,57,58].

### 3.4. Photosystem II (PS-II)-Inhibitors

PS-II inhibitors act by competitively binding to the plastoquinone binding site (Q_B_) on the D1 protein in the PS-II complex of the chloroplast [59]. This blockage disrupts photosynthesis since plastoquinone is vital for the electron transfer from PS-II to PS-I, and for generating nicotinamide adenine dinucleotide phosphate (NADPH) and ATP. The D1 protein is encoded by chloroplastic *psbA* gene, and hence, TSR to PS-II inhibitors can only be inherited maternally [60]. So far, 74 weed species have been reported to evolve resistance to PS-II inhibitors globally, via both TSR and NTSR mechanisms [13]. TSR to PS-II inhibition as a result of point mutations in the Q_B_ binding site has been reported in several weeds, such as kochia [61] and wild radish [62].

NTSR to PS-II inhibitors have been documented in annual bluegrass [63], common ragweed [64], common waterhemp [65,66], Palmer amaranth [67,68], and wild radish [62]. In the majority of these cases, the metabolism of PS-II inhibitors was catalyzed by the enhanced activity of GST enzymes [69] and/or CYP450 enzymes [70]. For example, atrazine-resistant Palmer amaranth from Kansas was found to conjugate atrazine 24 times faster than the susceptible plants via enhanced GST-activity [67]. Similarly, the enhanced metabolism of atrazine was found in two common waterhemp populations from Illinois [10] and Nebraska [65]. In the atrazine-resistant common waterhemp from Nebraska, at 6 HAT, approximately 92% of the atrazine was found to be conjugated by GSTs, whereas 92% of atrazine was still retained as a parent compound in susceptible plants [65]. Involvement of a phi-class GST, i.e., AtuGSTF2, was identified in mediating atrazine resistance in common waterhemp from Illinois [66]. Application of GST-inhibitors like 4-chloro-7-nitro-1,2,3-benzoxadiazole (NBD-cl) prior to atrazine application in resistant common waterhemp has resulted in greater sensitivity to atrazine [10]. Similarly, pre-treatment with a CYP450 inhibitor, 1-aminobenzotriazole, has shown increased sensitivity to resistant rigid ryegrass to simazine [70]. Apart from increased metabolism, reduced absorption and translocation can also impart PS-II-inhibitor resistance. Reduced absorption, translocation and increased metabolism of atrazine were observed in a PS-II-inhibitor (atrazine, diuron, semicarbazone)-resistant annual bluegrass population where known mutations in the *psbA* gene were lacking [63].

### 3.5. Enolpyruvyl Shikimate-3-Phosphate Synthase (EPSPS)-Inhibitors

Glyphosate, a non-selective, broad-spectrum herbicide, inhibits EPSPS in the shikimate pathway by acting as a transition state analog of phosphoenolpyruvate (PEP), which is a substrate for EPSPS [71]. The shikimate pathway produces the aromatic amino acids tryptophan, tyrosine, and phenylalanine, which are vital for plant growth and development. Additionally, glyphosate can increase carbon flow to the shikimate pathway, resulting in a shortage of carbon for other essential pathways [72]. Currently, there are 44 weeds reported to have evolved resistance to glyphosate [13]. Many of these resistance cases are either by alteration in the target (*EPSPS*) gene [73,74] or amplification and over-expression of the target gene [75,76,77].

Reduced translocation of glyphosate to meristematic sinks has been reported as the most common NTSR mechanism [78,79,80]. This mechanism has been reported in Palmer amaranth [81,82,83], horseweed [84], hairy fleabane [84], Italian ryegrass [85], rigid ryegrass [74,86], common waterhemp [73], Johnsongrass [87,88], sourgrass [89], and giant ragweed [90]. Reduction in translocation has been attributed to the evolution of a transporter that sequesters glyphosate inside the plant vacuole, thus preventing it from reaching the chloroplast [78]. In glyphosate-resistant horseweed, more (>85%) glyphosate was present in the vacuole of the resistant compared to only 15% in the susceptible plants [80]. Such sequestration was irreversible at least up to several days following the glyphosate application [80,91,92]. Similar modified sub-cellular distribution of glyphosate was found in glyphosate-resistant *Conyza bonariensis* [79]. ABC transporter proteins have been proposed to sequester glyphosate via active glyphosate transport [78,93]. Through GS-FLX 454 pyrosequencing, an increased expression of several *ABC* transporter genes was found in glyphosate-resistant horseweed following glyphosate application [94]. However, the role of a specific gene or gene family mediating glyphosate sequestration resulting in NTSR is still unknown. The reduced uptake of glyphosate has also been shown to impart low-level resistance to glyphosate in several weeds, such as Palmer amaranth [81,83], sourgrass [89], and Johnsongrass [87]. An enhanced metabolism of glyphosate is another mechanism responsible for high tolerance to glyphosate and was observed in some biotypes of sourgrass [95], horseweed [96], and *Echinochloa colona* [97]. In sourgrass biotypes with a greater tolerance to glyphosate, more than 56% of glyphosate was metabolized into aminomethylphosphonic acid (AMPA), glyoxylate, and sarcosine at 168 HAT compared to 10% in susceptible biotypes [95]. Similar, rapid metabolism of glyphosate was observed in resistant horseweed populations where almost 100% of glyphosate metabolized into glyoxylate, sarcosine, and AMPA within 96 HAT [96]. Through RNA-seq analysis, an aldo-keto reductase (AKR) contig with greater expression and activity, exhibiting metabolic resistance to glyphosate was identified in an *Echinochloa colona* population from Australia [97]. Further, glyphosate metabolites, such as AMPA and glyoxalate, were also found in *Escherichia coli* expressing the *AKR* gene (*EcAKR4-1*), which was similar to the resistant *Echinochloa colona* plants [97].

### 3.6. 4-Hydroxyphenylpyruvate Dioxygenase (HPPD) and Carotenoid-Inhibitors

HPPD enzyme is required for catalyzing the conversion of 4-hydroxyphenylpyruvate (HPP) to 2,5-dihydroxyphenylacetate (homogentisate) to produce plastoquinone and tocopherols in the carotenoid biosynthesis pathway [98]. Plastoquinone is essential for the electron transfer from PS-II to PS-I and also as a co-factor of phytoene desaturase (PDS), required for carotenoid formation [99]. Hence, most of these herbicides inhibit carotenoid formation, ultimately resulting in photo-oxidation of chlorophyll molecules and lipid peroxidation of the cell membranes by forming singlet oxygen [100]. So far, NTSR to HPPD-inhibitors has been reported most often. However, TSR caused by higher *HPPD* gene and protein expression has been reported in mesotrione-resistant Palmer amaranth [99].

Enhanced metabolism was the primary NTSR mechanism reported in HPPD-inhibitors-resistant Palmer amaranth [99,101,102], common waterhemp [10,36,103,104,105], and rice barnyardgrass [106]. In mesotrione-resistant Palmer amaranth, more than 90% of mesotrione was metabolized at 24 HAT [99]. Rapid 4-hydroxylation, followed by glycosylation and a higher expression of certain CYP450 enzymes, were identified in tembotrione-resistant Palmer amaranth compared to the susceptible biotype [102]. Similarly, increased mesotrione metabolism via 4-hydroxylation of the dione ring was reported in mesotrione-resistant common waterhemp from Nebraska [105]. Pre-treatment with CYP450-inhibitors has been shown to increase the sensitivity of resistant common waterhemp populations to mesotrione [10,36,103]. CYP450s belonging to sub-family CYP81A were found to impart metabolic-resistance to clomazone in rice barnyard grass [106]. *Arabidopsis* lines transformed with CYP81A12, CYP81A21, CYP81A15, and CYP81A24 showed increased resistance to clomazone, indicating their involvement in metabolizing clomazone [106].

### 3.7. Protoporphyrinogen Oxidase (PPO)-Inhibitors

PPO inhibitors are important broad-spectrum herbicides that growers can use to control weeds resistant to ALS inhibitors and glyphosate [107]. PPO inhibitors impede the PPO enzyme, which is required for catalyzing the conversion of protoporphyrinogen IX to protoporphyrin IX in the last step of plant heme and chlorophyll biosynthesis [108,109]. The inhibition of the PPO enzyme leads to the accumulation of intermediates in the cytosol, which are photoactively oxidized, ultimately leading to the production of highly reactive oxygen species (ROS). These ROS attack lipids and proteins in cell membranes and cause lipid peroxidation, leading to plant death [110]. So far, 13 weeds have evolved resistance to PPO inhibitors [13]. The most common mechanism of resistance reported was a single amino acid deletion (Gly210) or substitution in the *PPX2* (e.g., Arg98Leu) gene [107].

NTSR-based PPO inhibitor resistance has been reported in two pigweed species: Palmer amaranth [111] and common waterhemp [112]. A common waterhemp population resistant to carfentrazone-ethyl lacked the presence of known mutations previously reported to confer TSR to PPO inhibitors but exhibited increased sensitivity to carfentrazone-methyl when pre-treated with malathion [112]. This suggests the possible involvement of CYP450 in conferring resistance to carfentrazone-ethyl in common waterhemp [112]. Similarly, the absence of known mutations was reported in fomesafen-resistant Palmer amaranth [111]. The same population was further found to be cross-resistant to flumioxazin, acifluorfen, and saflufenacil [113]. Involvement of both CYP450s and GSTs was reported to mediate fomesafen resistance in Palmer amaranth due to increased sensitivity when pre-treated with malathion or NBD-cl [111,113].

### 3.8. Photosystem I (PS-I)-Inhibitors

Paraquat is a non-selective, fast-acting herbicide that diverts electrons from PS-I, leading to the inhibition of photosynthesis. Paraquat accepts a single electron in order to generate a reduced cation radical, that on further reaction with oxygen, generates a superoxide ion [114]. In the presence of light, paraquat catalyzes the production of superoxide ions, which eventually form hydroxy radicals and result in lipid peroxidation [114,115]. Only a single NTSR mechanism, i.e., reduced translocation via vacuolar sequestration, has been reported in horseweed [84], hairy fleabane [84], rigid ryegrass [116], and Italian ryegrass [117]. The amount of paraquat present in the leaf protoplast of resistant and susceptible rigid ryegrass was estimated and a 2–3-fold higher retention of herbicide was found in leaves of resistant plants, indicating possible sequestration of the herbicide in vacuoles [116]. A similar mechanism of paraquat resistance was reported in Italian ryegrass [117]. However, the molecular basis of such sequestration is still unknown.

### 3.9. Very Long Chain Fatty Acid (VLCFA) Synthesis-Inhibitors

VLCFA inhibiting herbicides are known to affect several steps in the elongation of the carbon chain of very long chain fatty acids [118]. VLCFAs are required for the formation of triacylglycerols, waxes, phospholipids, and complex sphingolipids, which are essential for various plant functions [119]. For instance, phospholipids and sphingolipids are required during cell division, as well as for maintaining membrane trafficking pathways [119,120,121]. The ever-increasing occurrence of ACCase and ALS-inhibitor resistance has led growers to rely more on VLCFA inhibitors, which are important pre-emergence herbicides for controlling grasses.

NTSR mechanisms to very long chain fatty acid (VLCFA) inhibitors have been studied in ryegrass populations from Australia [122], U.K., and France [123], as well as Palmer amaranth from Arkansas [124]. Metabolic resistance to pyroxasulfone was reported in a rigid ryegrass population from Australia, where approximately 88% of parental applied herbicides was metabolized within 24 HAT [122]. Pyroxasulfone metabolites were formed via glutathione conjugation and two putative *GST* genes were 2–6-fold constitutively overexpressed in resistant ryegrass populations [122]. Interestingly, continuous sub-optimal application of pyroxasulfone can rapidly select for resistant biotypes of rigid ryegrass [125]. Application of herbicides at sub-optimal doses can favor the selection of several minor resistance alleles and facilitate their accumulation in cross-pollinating weeds like rigid ryegrass, leading to rapid evolution of polygenic NTSR [126,127]. Moreover, such selection can promote the evolution of cross-resistance to other VLCFA inhibitors like prosulfocarb and triallate [128]. In flufenacet-resistant ryegrass populations from the U.K. and France, enhanced metabolism due to conjugation by GSTs was reported [123]. Similarly, enhanced metabolism of flufenacet was observed in resistant blackgrass [129]. Recently, resistance to *s*-metolachlor was documented in Palmer amaranth from Arkansas. A reduction in root growth was observed when the resistant accessions were kept for germination in agar solution containing a GST-inhibitor, NBD-cl, indicating the role of GSTs in mediating the resistance [124].

## 4. Influence of Environmental Factors on NTSR Mechanisms

NTSR mechanisms can be affected by changes in environmental conditions [130,131]. Factors like the mode of action of herbicides and the physiology of weed species can contribute significantly in the alteration of NTSR under different environmental conditions. Both herbicide-resistant and susceptible biotypes have shown increased and decreased tolerance to herbicides under different environmental conditions. NTSR mechanisms are suspected to develop gradually in response to biotic and abiotic stresses, which enable them to adapt to the growing conditions [132]. Changing environmental conditions can seriously affect herbicide efficacy and favor the selection of more tolerant biotypes. Hence, information on how NTSR mechanisms behave in different weed species in varying environmental conditions can be very crucial to mitigate such selection. The effect of environmental conditions, such as temperature, CO_2_ concentration, and relative humidity, on NTSR mechanisms has been studied in several weed species. Altered temperature regimes were shown to impact herbicide absorption [133,134,135,136], translocation and sequestration [133,134,136,137,138,139,140,141], and metabolism [30,139,142]. For example, exposure to high temperature was found to reduce pinoxaden sensitivity of grass species, such as *Brachypodium hybridum* [142]. A higher level of inactive glucose-conjugated pinoxaden metabolites in these grasses was observed under high- versus low-temperature conditions [142]. Such increased detoxification of pinoxaden in *Brachypodium hybridum* was associated with a possible increased enzymatic activity of reactive oxygen species scavengers [30]. In another study, the suppression of vacuolar sequestration of glyphosate at low temperature was found to result in the increased sensitivity of glyphosate-resistant horseweed [137]. Recently, poor control of kochia due to the reduced absorption of glyphosate and translocation of dicamba at high temperature was reported [133]. Decreased efficacy of mesotrione in controlling Palmer amaranth due to rapid metabolism at high temperature was documented [139]. Conversely, improved 2,4-D efficacy at high temperature due to increased translocation was found in both glyphosate-resistant and -susceptible common and giant ragweed [134]. Additionally, increased absorption of glyphosate in common ragweed, and increased absorption and translocation of glyphosate in giant ragweed, improved glyphosate efficacy at high temperature [134]. Apart from temperature, changes in CO_2_ concentrations can affect herbicide translocation and sequestration in weeds [140,143]. In horseweed and lambsquarter, an increase in glyphosate translocation was found at elevated CO_2_ levels and increased temperature, leading to a reduced glyphosate sensitivity [140]. Similarly, in *Echinochloa colona*, high CO_2_ and increased temperature reduced the efficacy of cyhalofop-butyl by decreasing translocation [143]. Altering relative humidity (RH) can also affect herbicide translocation in pigweeds [144]. For instance, in Palmer amaranth, redroot pigweed, and common waterhemp, a higher translocation of glufosinate was found at high, compared to low, RH [144]. These findings indicate the need to further elucidate and evaluate the impact of environmental conditions on the sensitivity of weeds to herbicides to slow evolution of herbicide resistance.

## 5. Coexistence of TSR and NTSR Mechanisms

Numerous cases of TSR have been reported as a result of single nucleotide polymorphisms resulting in amino acid substitutions in the target sites of several herbicides, such as PS-I, ALS, and ACCase inhibitors, and glyphosate. Novel mechanisms of TSR in weeds, such as the deletion of codons, leading to PPO-inhibitor resistance in common waterhemp and Palmer amaranth [107,145], as well as gene amplification-based resistance to glyphosate [75,77] and ACCase inhibitors [146], were reported. These findings will help in identifying the precise genetic elements involved in the evolution of TSR in resistant weeds. Similarly, recent advances have also helped to understand the physiological and molecular basis of NTSR in weed species. Interestingly, several cases of coexistence of these mechanisms have been reported. For instance, ALS-inhibitor-resistant water chickweed [33] and barnyard grass [147], ACCase-inhibitor-resistant *Vulpia bromoides* [148], Italian ryegrass [19], short awn foxtail [41], PS-II inhibitor-resistant wild radish [62], EPSPS-inhibitor-resistant rigid ryegrass [74], common waterhemp [73], HPPD-inhibitor-resistant Palmer amaranth [99], and microtubule inhibitor-resistant rigid ryegrass [34] have been identified with TSR and NTSR mechanisms in the same populations. Therefore, if TSR mechanisms are found to contribute to herbicide resistance, it is also necessary to investigate the NTSR mechanisms and vice versa. Although deciphering both types of resistances in the same weed species can be challenging, understanding the coexistence of TSR and NTSR mechanisms for the same herbicide is valuable for predicting possible cross-resistance to other herbicides, thereby assisting in management of resistance.

## 6. Conclusions and Prospects

Weed management practices can impact the mechanisms by which weeds evolve resistance to herbicides. Additionally, a key aspect in predicting the evolutionary trajectory of herbicide-resistant traits is understanding the mechanism(s) of herbicide resistance. More importantly, understanding the relationship between the weed management tactics and their influence on evolutionary mechanisms (TSR or NTSR) that determine herbicide resistance in weed species will help to formulate effective future strategies to manage these increasingly problematic weeds. It has been proposed that a lower rate of herbicides result in the evolution of polygenic traits, whereas high herbicide doses may favor monogenic target-site-based resistances [127]. Likewise, Gressel [149] proposed that suboptimal herbicide use rates can result in the evolution of polygenic herbicide resistance. Understanding the type of selection pressure leading to the evolution of NTSR mechanisms, especially metabolic resistance, is extremely valuable and needed to sustain the limited herbicide portfolio and develop integrated weed management strategies.
